# Management of patients at very high risk of osteoporotic fractures through sequential treatments

**DOI:** 10.1007/s40520-022-02100-4

**Published:** 2022-03-24

**Authors:** Elizabeth M. Curtis, Jean-Yves Reginster, Nasser Al-Daghri, Emmanuel Biver, Maria Luisa Brandi, Etienne Cavalier, Peyman Hadji, Philippe Halbout, Nicholas C. Harvey, Mickaël Hiligsmann, M. Kassim Javaid, John A. Kanis, Jean-Marc Kaufman, Olivier Lamy, Radmila Matijevic, Adolfo Diez Perez, Régis Pierre Radermecker, Mário Miguel Rosa, Thierry Thomas, Friederike Thomasius, Mila Vlaskovska, René Rizzoli, Cyrus Cooper

**Affiliations:** 1 MRC Lifecourse Epidemiology Centre, University of Southampton, Southampton, UK; 2 WHO Collaborating Centre for Public Health Aspects of Musculoskeletal Health and Aging, Liège, Belgium; 3 Department of Public Health, Epidemiology and Health Economics, University of Liège, CHU Sart Tilman B23, 4000 Liège, Belgium; 4 Biochemistry Department, College of Science, King Saud University, 11451 Riyadh, Kingdom of Saudi Arabia; 5 Division of Bone Diseases, Department of Medicine, Faculty of Medicine, Geneva University Hospitals, University of Geneva, Geneva, Switzerland; 6 F.I.R.M.O, Italian Foundation for the Research on Bone Diseases, Florence, Italy; 7 Department of Clinical Chemistry, University of Liege, CHU de Liège, Liège, Belgium; 8 Center of Bone Health, Frankfurt, Germany; 9 Philipps-University of Marburg, Marburg, Germany; 10 International Osteoporosis Foundation, Nyon, Switzerland; 11 NIHR Southampton Biomedical Research Centre, University of Southampton and University Hospital Southampton NHS Foundation Trust, Southampton, UK; 12 Department of Health Services Research, Care and Public Health Research Institute (CAPHRI), Maastricht University, Maastricht, The Netherlands; 13 NDORMS, University of Oxford, Windmill Road, Oxford, UK; 14 Mary McKillop Institute for Health Research, Australian Catholic University, Melbourne, Australia; 15 Centre for Metabolic Bone Diseases, University of Sheffield Medical School, Beech Hill Road, Sheffield, UK; 16 Department of Endocrinology, Ghent University Hospital, Gent, Belgium; 17 University of Lausanne, UNIL, CHUV, Lausanne, Switzerland; 18 Faculty of Medicine, University of Novi Sad, Novi Sad, Serbia; 19 Clinical Center of Vojvodina, Clinic for Orthopedic Surgery, Novi Sad, Serbia; 20 Department of Internal Medicine, Hospital del Mar-IMIM, Autonomous University of Barcelona and CIBERFES, Instituto Carlos III, Madrid, Spain; 21 Department of Diabetes, Nutrition and Metabolic Disorders, Clinical Pharmacology, University of Liege, CHU de Liège, Liège, Belgium; 22 Faculty of Medicine, University of Lisboa, Lisbon, Portugal; 23 Department of Rheumatology, Hôpital Nord, CHU Saint-Etienne, Saint-Etienne, France; 24 INSERM U1059, Université de Lyon, Université Jean Monnet, Saint-Etienne, France; 25 Medical Faculty, Department of Pharmacology and Toxicology, Medical University Sofia, Sofia, Bulgaria; 26 NIHR Oxford Biomedical Research Centre, University of Oxford, Oxford, UK

**Keywords:** Osteoporosis, Epidemiology, Imminent, Fracture, Anabolic, Antiresorptive

## Abstract

Osteoporosis care has evolved markedly over the last 50 years, such that there are now an established clinical definition, validated methods of fracture risk assessment and a range of effective pharmacological agents. Currently, bone-forming (anabolic) agents, in many countries, are used in those patients who have continued to lose bone mineral density (BMD), patients with multiple subsequent fractures or those who have fractured despite treatment with antiresorptive agents. However, head-to-head data suggest that anabolic agents have greater rapidity and efficacy for fracture risk reduction than do antiresorptive therapies. The European Society for Clinical and Economic Aspects of Osteoporosis, Osteoarthritis and Musculoskeletal Diseases (ESCEO) convened an expert working group to discuss the tools available to identify patients at high risk of fracture, review the evidence for the use of anabolic agents as the initial intervention in patients at highest risk of fracture and consider the sequence of therapy following their use. This position paper sets out the findings of the group and the consequent recommendations. The key conclusion is that the current evidence base supports an “anabolic first” approach in patients found to be at very high risk of fracture, followed by maintenance therapy using an antiresorptive agent, and with the subsequent need for antiosteoporosis therapy addressed over a lifetime horizon.

## Introduction

Osteoporosis presents a massive, and inexorably increasing, burden on health and social care, at the levels of patients, healthcare providers and policymakers [1–5]. Osteoporotic fractures are associated with reduced quality of life and significant morbidity, mortality and utilisation of healthcare resources [4]. Between 2010 and 2040, the number of individuals at high risk of fracture is predicted to double worldwide, with the largest increases (relative increases of twofold or greater) expected in Africa, Latin America and Asia [6]. In Europe, the recent ScoreCard for OsteoPorosis in Europe (SCOPE) collaboration has estimated that by 2034, 5.34 million individuals will be affected by osteoporotic fractures in the European Union plus the UK and Switzerland (EU 27 + 2 countries), an increase of 1.06 million (24.8%) from 2019 [4].

Despite the fact that osteoporosis is a well-recognised problem with a choice of affordable and widely available treatments (particularly for the health economies of high-income countries), a large treatment gap exists. In the EU27 + 2, 71% of women (15 of 21 million) likely to warrant treatment for osteoporosis are left without appropriate medication [4]. An International Osteoporosis Foundation report suggested that this was due to a variety of challenges in case finding and management, a lack of public awareness (including misconceptions regarding the benefit–risk balance of treatment), and government and health system issues (such as difficulty in accessing reimbursement for osteoporosis treatments, and lack of prioritisation of fracture prevention in national health policy) [7].

Whilst there is clearly a major issue with treating patients at all, there is also increasing evidence to suggest that stratification of treatment according to baseline fracture risk may permit targeting of the most effective treatments to patients at the highest fracture risk [8]. Such a strategy would ensure greatest rates of fracture risk reduction in those most likely to fracture and thus contribute to addressing the current treatment gap as well as maximising benefits for the most vulnerable individuals. Some treatments for osteoporosis, for example oral bisphosphonates, menopausal hormonal therapy (MHT) and selective oestrogen receptor modulators (SERMs) have suboptimal efficacy; studies of goal-directed treatment in osteoporosis have highlighted difficulties in meeting treatment goals with such therapies in the highest fracture risk patients [9]. Consequently, the IOF and European Society for Clinical and Economic Aspects of Osteoporosis, Osteoarthritis and Musculoskeletal Diseases (ESCEO) published guidance for the diagnosis and management of osteoporosis in 2019, with subsequent recommendations on treatment stratification in 2020 [10], stating that, in patients at the highest risk of fracture, treatment initiation with an anabolic (bone-forming) agent such as teriparatide, abaloparatide or romosozumab, followed by an antiresorptive to maintain the gains in bone mineral density, appears now a highly appropriate strategy to achieve a rapid and sustained reduction in fracture risk [11, 12]. This recommendation has strong evidential support from recent studies comparing anabolic with antiresorptive medications, demonstrating a more rapid and greater fracture risk reduction with the former, compared with antiresorptive treatments alone [13–16].

Implementation of such guidance, however, presents some challenges: How should a patient at very high fracture risk be identified? Which anabolic drug should be chosen (and likely not just on the basis of efficacy and clinical profile, but dependent upon local licensing and reimbursement considerations)? What should be the duration of anabolic therapy, and what should be the duration of subsequent antiresorptive therapy? Can a patient be cycled between anabolic and antiresorptive treatment multiple times? Is sequential therapy with an anabolic first a cost-effective way of allocating resources?

To address these questions, a working group convened by the European Society for Clinical and Economic Aspects of Osteoporosis, Osteoarthritis and Musculoskeletal Diseases (ESCEO) met to review current data on these topics. The group comprised 25 experts from 13 countries with expertise covering osteoporosis, rheumatology, geriatrics, clinical chemistry, epidemiology, public health, health services research, health economics and drug safety. This consensus report summarises the working group’s assimilation of the current literature and consequent recommendations regarding strategies for the identification and sequential treatment, through anabolic and antiresorptive medications, of patients at very high fracture risk.

## Approaches to risk stratification

### Introduction

Several approaches have been developed with which to identify individuals at very high fracture risk. These range from criteria based on clinical scenarios, to predictive algorithms derived using population registries, for example with machine learning techniques, and modifications to existing algorithms such as the FRAX^®^ fracture risk assessment calculator. An important nuance in the interpretation of the outputs from such methods is that of the time horizon considered [17]. The concept of “imminent” fracture risk has been discussed increasingly widely but has a variety of meanings in different contexts. For example, several studies have examined predictors of risk over a subsequent two-year period with the inference that these predict a fracture risk which is “imminent” given the short time horizon [18]. However, as will be discussed below, there are only a minority of clinical risk factors which raise fracture risk in the short much more than in the long-term. Recent prior fracture and supraphysiologic corticosteroid treatment are examples, but risk factors associated with higher short-term risk are usually also associated with high long-term risk [17]. A second characterisation of “imminent fracture risk” is that directly linked to the transient markedly increased risk of a subsequent fracture immediately after an index event [19]. This will be discussed in more detail in the section on the FRAX tool below and represents one route into a very high fracture risk, alongside other combinations of risk factors.

### Cohort-derived methods

Different audiences, for example, policy makers undertaking evaluation of expensive new antiosteoporosis therapies; clinicians deciding which patients are at very high risk and are suitable for the most potent treatments and tightest monitoring; and patients considering whether or not they wish to take an antiosteoporosis medication, are likely to require different characteristics in an assessment of fracture risk. Policy makers may prefer accurate, complicated (requiring large volumes of input data), evidence-based models; clinicians require validity for their individual patient, with readily available inputs and easily actionable outputs; patients may prefer personalised, short-term outcomes, which are relatable to other health and lifestyle-related risks to help inform decision-making.

Aside from FRAX (discussed in more detail below), other cohort-derived risk calculators are available, including Q-Fracture (https://qfracture.org/) and Garvan (https://www.garvan.org.au/promotions/bone-fracture-risk/calculator/) fracture risk calculators [20–22]. Q-Fracture, though not widely used, is integrated into some Primary Care patient management systems in the UK and allows one to alter the duration of risk, from 2 years, to 5 years, to 10 years, apparently using a simple arithmetic approach. Garvan provides the option for a 5-year risk output, though again this is just half the calculated 10-year risk—as discussed previously, imminent fracture risk is important and there is not a linear accrual of fractures over a 10-year timeframe, with fracture risk much higher in the first two years post an index fracture event than over the remaining eight years of a 10-year time horizon [19].

Multi-cohort approaches have been used more recently, aiming to characterise 1- to 2-year fracture risk. A study led by Prieto-Alhambra et al. used primary care data in patients at high fracture risk from the UK Clinical Practice Research Datalink (CPRD) (*n* = 83,000 linked to Hospital Episode Statistics in England), the Catalonian Information System for Research in Primary Care (SIDIAP) (n = 51,000, linked to regional hospital data), and the Danish Health Registry (DHR) (*n* = 509,000) (linked to the Danish national register of diagnostic codes for all inpatients and outpatients). The authors were able to demonstrate that individuals with an incident fracture (at any site, excluding the skull, face or digits) were at increased risk of subsequent hip, clinical spine, humerus or distal forearm fractures (major osteoporotic fractures), with a 2-year incidence rate per 10,000 person-years of 22.0 (95% CI 21.2–22.8) in the UK CPRD; 24.0 (95% CI 23.0–25.0) in Spain (SIDIAP); and 36.9 (36.5–37.3) in Denmark (DHR). Such differences between countries may be explained by data collection methods as well as true differences in population level fracture risk [23]. The study also examined major osteoporotic fracture risk after index hip fracture at 1-year follow-up in men and women aged over 50 years; incidence rates were low, however, and provided a 1-year risk of around 2% in CPRD/SIDIAP and 5% in DHR [24]. The incident fragility fracture prediction model (in which the model parameters are freely available) demonstrated good performance for hip fracture at 1 year in Spain, Denmark and the UK, and calibration was good across all three countries, with the authors concluding that such imminent fracture risk prediction models could be used to precisely identify patients at high imminent risk of fracture, thereby informing antiosteoporosis treatment selection [25].

In a Dutch study of 4140 postmenopausal women with a known fracture history, after a first fracture 23% of all subsequent fractures occurred within 1 year and 54% within 5 years. When calculated from time of first fracture, the relative risk (RR) of subsequent fracture was 2.1 (95% CI 1.7–2.6) and remained increased over 15 years. This study demonstrated elevated imminent fracture risk, as, when calculated for specific time intervals after a first fracture, the RR was 5.3 (95% CI 4.0–6.6) within 1 year, 2.8 (95% CI 2.0–3.6) within 2–5 years, 1.4 (95% CI 1.0–1.8) within 6–10 years and 0.41 (95% CI 0.29–0.53) after > 10 years [26].

The Swedish national patient register has also been used to evaluate the cumulative incidence of new fractures. In a study of 35,146 women with a mean age of 73.8 years and an index fracture in 2013, cumulative incidence of a new fracture over 2 years was 11%, with fracture location influencing the incidence and type of subsequent fracture. The risk of second fracture was highest in index clinical vertebral (18%) and hip fractures (14%), and despite high re-fracture rates, low treatment rates were observed across all fracture types and age groups [27]. Using a similar design, amongst insurance scheme registrants in Germany with an index fracture of the hip, vertebra, forearm or upper arm (18,354 male and female patients, mean age 77 years), 15% sustained a subsequent fracture during a shorter, 1-year follow-up period, with those with an index vertebral fracture again being at highest risk (18%) and highlighted as a group for urgent antiosteoporosis treatment [28].

### Machine learning approaches

**Fig. 1:**
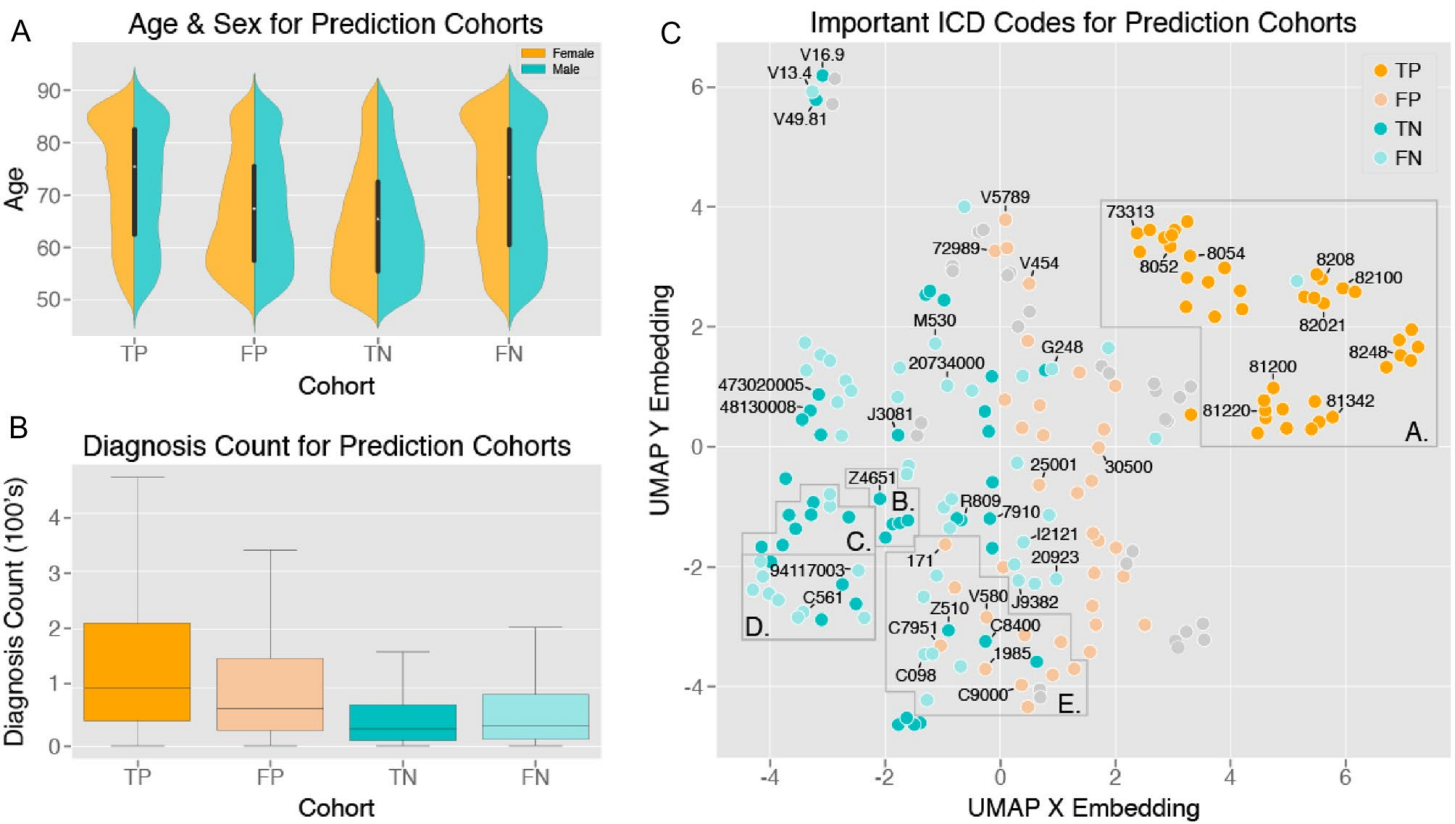
Crystal Bone: Use of Machine Learning driven methods for fracture prediction. Crystal Bone used techniques applied in natural language processing to screen electronic healthcare records from a US population (Optum), covering 91 million patients to predict first and second fracture at the spine, pelvis, clavicle, humerus, radius, ulna, hip, femur, tibia, fibula, and ankle. Sequences of ICD codes were used as inputs to implement two distinct frameworks: (1) ICD code vectorization and long short-term memory networks, and (2) patient-level vectorization and extreme gradient boosting decision trees. The figure shows exploration of model interpretability by comparison of various characteristics of the input data for the 4 prediction cohorts of the confusion matrix (*FN* false negative, *FP* false positive, *TN* true negative, *TP* true positive). UMAP: uniform manifold approximation and projection (this allows encoded vectors to be projected onto a 2D space for dimension reduction). ICD codes predictive of future fracture (TP) include, for example 73,313 (collapsed vertebra), 81,200 (closed fracture of upper humerus), 81,342 (closed fracture of distal radius), 82,100 (closed fracture of femur). Reproduced with permission from [29]. https://www.jmir.org/2020/10/e22550/

A novel approach to the identification of patients at high fracture risk over a short time frame uses machine learning technology, applying deep learning to sequential patient data. *Crystal Bone* is a machine learning approach using longitudinal data contained in electronic health records, and was developed using subsets of the United States’ Optum (UnitedHealth Group) deidentified electronic health data record set covering 91 million patients from over 140,000 providers, from which patients with osteoporosis, fractures or bone related medications were included [29]. Using data from over 1 million patients, the Crystal Bone algorithm was developed, leveraging techniques typically applied in natural language processing (to understand large volumes of textual data), focusing on International Classification of Diseases (ICD) codes (Fig. 1). The goal was to evaluate the ability of these language processing-based models to learn patterns associated with increased 2-year fracture risk. The model was shown to accurately predict 1–2-year fracture risk for patients aged over 50 years, with an area under the receiver-operating characteristics curve (AUROC) 0.81, representing an improvement over baseline models and FRAX 10-year risk, though the authors acknowledged that direct comparisons cannot be made. Similarly, the Danish Fracture Risk Evaluation Model (FREM) aimed to identify conditions for inclusion in a fracture prediction model for automated case finding of high risk individuals for hip or major osteoporotic fracture (MOF). The whole of the Danish population aged over 45 years was studied (almost 2.5 million individuals), incorporating all hospital diagnoses from 1998 to 2012 with MOFs in 2013 as the primary outcome. Backward selection on ICD-10 codes (International Classification of Diseases and Related Health Problems, 10th Revision) by logistic regression was used to develop an age-adjusted and sex-stratified model in development and validation datasets. The FREM included 38 and 43 risk factors for MOF in women and men, respectively, and produced receiver-operating characteristic (ROC) curves with an area under the ROC curve (AUC) of 0.750 (95% CI 0.741–0.795) and 0.752 (95% CI 0.743–0.761) for women and men, demonstrating good accuracy, with even higher accuracy demonstrated for hip fractures [30]. The model has also recently been shown to generate similar results when using a 5-year look-back as opposed to applying data from a 15-year period, implying it may be applied to improved identification of individuals at high imminent risk of fracture [31].

Clearly, there may be problems in applying these findings to other populations due to lack of generalisability of both the patient population and the available variables, and questions arise as to how these approaches can be implemented in other countries. The Danish FREM algorithm, for example, significantly overestimated hip fracture risk when applied to an independent clinical population from Manitoba, Canada, indicating the need for recalibration [32]. However, in health care systems where such rich data and machine learning technologies are available, this approach may indeed be a practicable future direction of travel, and could help with the identification of patients at very high risk of fracture, perhaps as a first population level healthcare provider “screen” for those who might warrant further assessment.

### Expert consensus and BMD-based approaches

Expert consensus around clinical scenarios and/or BMD represents another route to the definition of very high fracture risk and of imminent fracture risk. Whilst the ESCEO and the IOF, in the European guidelines, recommend FRAX-based approaches, as previously described [11, 12, 10], the US Endocrine Society use a prior fracture and T-score based approach, defining very high risk as an individual with multiple spine fractures and a T-score of ≤ − 2.5 [33]. The UK Scottish SIGN guidelines employ a similar approach, defining severe osteoporosis as the presence of 1 severe or 2 or more moderate vertebral fractures with a T-score of ≤ − 1.5, or a lumbar spine T-score of ≤ − 4, irrespective of fracture [34].

Consistent with the recent ESCEO-IOF and UK NOGG approaches [35], Swiss guidelines define very high fracture risk as when an individual’s 10-year probability of major osteoporotic fracture according to FRAX is at least 20% (1.2 ×) above the absolute risk intervention threshold at any age. High imminent risk is defined as 10% within 2 years, characterised by an age threshold of 65 years or older, and a prior fracture of major osteoporotic fracture (hip, clinical vertebral fracture, humerus, pelvis or radius) [36]. Recent operational guidance in the UK has taken a similar approach but with a 10-year time horizon suggesting that very high fracture risk, incorporating the notion of high imminent risk in the shorter term, may be captured by the presence of a recent fracture and a 10-year FRAX major osteoporotic fracture probability of 30% more [37].

Many similarities and many differences are present within these expert consensus guidelines. All agree that recent fractures should be defined as within the past 2 years. They differ by the use of DXA T-scores in some, versus FRAX thresholds in others (which may be fixed, or age related), and they also differ in their definitions of major osteoporotic fracture. Some include the distal forearm and some do not; such inconsistencies in expert recommendations may not help the osteoporosis field in general, supporting the need for a more unified approach.

### *FRAX*^*®*^* and imminent fracture risk*

The FRAX fracture risk assessment tool, in use since 2008, integrates information on 10 different clinical risk factors, with or without BMD measurement, generating 10-year probabilities for major osteoporotic fracture (hip, clinical vertebral, proximal humerus and distal forearm) and for hip fracture (https://www.sheffield.ac.uk/FRAX/) [38]. The clinical risk factors were chosen on the basis of intuitive linkage to fracture risk and ready clinical availability following a series of meta-analyses of prospective cohort studies from Europe, North America, Asia and Australia including nearly 45,000 individuals, and have subsequently been validated in other cohorts. A unique feature of the FRAX algorithm is the integration of risk of death with risk of fracture, to yield a 10-year probability of fracture, which incorporates not just fracture risk, but also the competing hazard of death. At older ages where predicted survival is less than 10 years, the output fracture probability reflects a remaining lifetime horizon [39]. At the time of writing, 81 FRAX models are available in 73 countries covering around 80% of the world population, and the tool is used in over 100 guidelines worldwide [11, 40–42]. Thresholds have been devised in many countries to advise clinicians on when to prescribe antiosteoporosis treatment (intervention thresholds) [41]. Given that most assessment guidelines recommend treatment in postmenopausal women with a prior fragility fracture, the European recommendations include age-dependent intervention thresholds which reflect the age-specific fracture probability equivalent to a woman of average BMI with a prior fragility fracture, no additional risk factors, and without knowledge of BMD [11]. In European countries (and indeed many others), around the intervention threshold, assessment of bone mineral density is recommended to determine whether a person lies above the threshold (at which point they would be recommended treatment) or below the threshold (at which point they would be recommended lifestyle advice) [11].

**Fig. 2:**
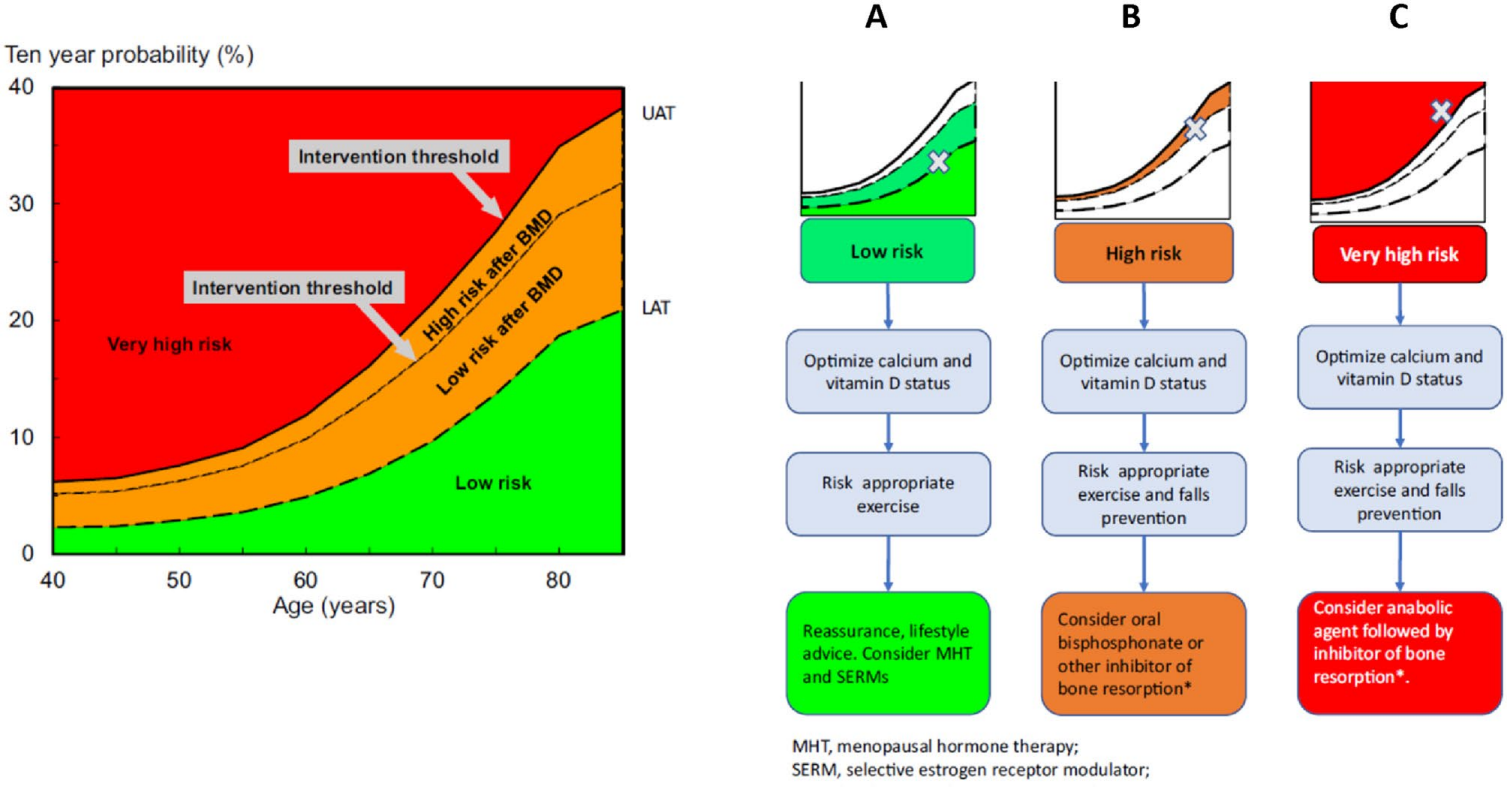
The characterisation of fracture risk according to FRAX major osteoporotic fracture probability in postmenopausal women. Initial risk assessment is performed using FRAX with clinical risk factors alone. FRAX probability in the red zone indicates very high risk, in which pathway C may be appropriate (anabolic agent followed by an inhibitor of bone resorption). FRAX probability in the green zone suggests low risk, in which pathway A should be followed, with advice to be given on lifestyle, calcium and vitamin D nutrition and menopausal hormonal treatment considered. FRAX probability in the orange zone (intermediate, between the upper assessment threshold, UAT, and lower assessment threshold, LAT) should be followed by BMD assessment and recalculation of FRAX probability including femoral neck BMD. After recalculation, risk may, therefore, be in the red zone (very high risk), orange zone (high risk, pathway B, which suggests initial antiresorptive therapy), reproduced with permission from [10].

With the increasing evidence for more rapid and greater magnitude of therapeutic effect for anabolic agents compared with antiresorptives (discussed in more detail in the next section), the IOF and ESCEO have recommended that individuals eligible for treatment be dichotomised into those at high risk and those at very high risk of fracture [10]. In this way, patients at very high risk of fracture can be directed to the more expensive but more efficacious anabolic therapy first [13–16], whilst those at high risk can be directed to an antiresorptive agent such as a bisphosphonate (Fig. 2).

Consistent with the age-dependent approach to the intervention threshold, in the IOF-ESCEO approach, very high risk can be defined as a fracture probability that lies above the upper assessment threshold (1.2 times the intervention threshold) after a FRAX assessment, with or without the inclusion of BMD, i.e. where BMD testing is unavailable, the same probability threshold can be used [43]. A similar approach has been applied nationally in the UK National Osteoporosis Guideline Group recommendations [35], with the threshold adapted to incorporate the constant probability threshold above the age of 70 years in this hybrid setting [44]. The next question to address is what attributes and clinical risk factors are associated with FRAX probabilities in low, high and very high fracture risk categories. In this setting, it is apparent that the presence of a single clinical risk factor rarely leads to very high fracture risk categorisation but a combination of risk factors, particularly older age, recent fracture and glucocorticoid use, more frequently result in this high fracture risk outcome [35].

As introduced earlier in this article, the trajectory of risk associated with the recent prior fracture appears to be a particularly important, but by no means exclusive, contributor to very high fracture risk categorisation. To this end, several studies have demonstrated that fracture risk is acutely elevated immediately after an index fracture and that this elevated risk wanes over the succeeding 2 years (this transiently elevated risk also can be termed “imminent” risk), but does not return to baseline and subsequently increases with age [26, 46, 47]. Thus, a fracture at any time in the past is associated with increased risk of an incident fracture event, but an index fracture is associated with a marked excess fracture risk over and above this in the next 2 years [17, 19]. This pattern has been most comprehensively assessed in the Iceland Reykjavík cohort [19, 45], and further data from the Reykjavik Study have shown that, in individuals who sustained a recurrent fracture, between 31 and 45% of fractures occurred within one year of the first (sentinel) fracture, depending on the fracture site [19]. Further work using this cohort has demonstrated that the transient risk increase following an index fracture is of great enough magnitude to materially alter the 10-year probability of fracture generated by the FRAX tool [19]. Importantly, the currently available tool does not incorporate recency of fracture, or indeed a different risk associated with different fracture sites and therefore will underestimate 10-year fracture probability in the context of a prior fracture in the last 2 years. To address this situation, multipliers specific to age, sex and fracture site have been generated to enable the physician to accommodate the excess risk associated with recency and particular fracture types [48]. The multiplier decreases with age, partly because of the competing effect of mortality with which recency is also associated, but because fracture probability is so strongly dependent upon age, the final adjusted absolute probability is almost always greater at older compared with younger ages [48]. It is also apparent that the magnitude of the absolute fracture probability is always greater when viewed over a 10-year, than over a 2-year, time horizon [49]. Development of a platform enabling the easy incorporation of the multiplier as a modifier of the FRAX calculator online is ongoing and will be available as FRAX_PLUS_. A key advantage of this approach is that recency and site of fracture, along with other modifiers of FRAX probability, for example dose of glucocorticoids, can be used to modify FRAX probability in a way that is immediately interpretable in the context of current national guidelines which are based on 10-year FRAX probability. The limitation of calculators and algorithms estimating fracture risk over the next 2 years is that, at present, there is generally no guideline infrastructure through which the outputs can be directly incorporated into clinical practice and there are few data to support their generalisability into other country settings. On a practical note, the key message in terms of fracture recency is that a fracture event requires urgent assessment of fracture risk and intervention with antiosteoporosis medications.

### Summary: fracture risk assessment

In summary, it is apparent that there are several different mechanisms for the identification of individuals at very high risk of fracture. These differ in their approach with the majority addressing fracture risk over a 2-year time horizon, terming this concept “imminent” fracture risk. Importantly the majority of such studies have not compared the predictive value over 2 years with that over 10 years and as such, it is very likely that being identified as at high risk in the next 2 years confers high risk over the next 10 years and indeed over the remaining lifetime [17, 49]. The most thoroughly characterised approach is that using the FRAX calculator linked with age-dependent intervention thresholds to identify individuals at high and very high fracture risk [48]. Importantly, whatever method is used in whatever context, the goal is to identify individuals at very high fracture risk because the higher the fracture risk the more likely there will be a fracture in the next few years (i.e. the more imminent the risk) and the more urgent is the need for treatment which is highly effective and rapid acting. Having established the different ways in which individuals at very high fracture risk may be identified, in the next section, we address the potential approaches to anabolic first therapy.

## Approaches to sequential therapy

### The anabolic first approach in very high fracture risk patients

There is increasing evidence to suggest that the anabolic agents for osteoporosis, teriparatide, abaloparatide, and romosozumab are superior in efficacy, and rapidity of action, to the antiresorptive agents in their ability to increase bone mineral density and prevent fractures [13–16]; these benefits must be maintained, however, by following the anabolic with an antiresorptive drug [14, 50, 51]. Indeed, there is also evidence to suggest that the sequence of treatment given to a patient with osteoporosis is important, such that an anabolic agent given prior to an antiresorptive agent is more efficacious than the opposite sequence [52, 53]. Whether this observation is generalisable across all anabolics is currently unknown, but based on existing evidence and understanding of mechanisms of action, it is likely to hold. Such agents are given over a relatively short time interval of one to two years, but the fact that BMD can be maintained with an inhibitor of bone resorption is extremely important in terms of fracture risk reduction. If we take the example of an anabolic agent (in a hypothetical population) that reduced the hip fracture risk by 70% (relative risk reduction, RRR = 70%), the anabolic agent, given for 18 months, then followed by an antiresorptive to maintain the effect for a total of 10 years, might be expected to save 33.8 hip fractures/1000 patient years in women aged 70 years with a recent fragility fracture [15]. In contrast, an antiresorptive (in the same hypothetical population) that reduced the hip fracture risk by 40% (RRR = 40%) followed by an anabolic regimen for the last 18 months of a 10-year treatment would save only 20.0 hip fractures/1000 patient years [10]. This alters the paradigm for the use of anabolic therapies, going beyond their current widespread use as “salvage therapy” when all other treatment has failed, to the notion of first-line anabolic treatment. In turn, this new paradigm suggests the need for clinicians to be able to identify the individuals who would most benefit from an anabolic therapy. The evidence for the efficacy of bone-forming agents in terms of BMD gained and fractures prevented is summarised below.

### Teriparatide

Teriparatide, recombinant human parathyroid hormone amino-terminal fragment, preferentially stimulates osteoblasts to produce new bone tissue, thereby increasing bone mass and strength [54] and has been shown to reduce vertebral and non-vertebral fractures in post-menopausal women with established osteoporosis at a daily dose of 20 µg/day SC [55]. The Fracture Prevention Trial in 2001 demonstrated the sustained effect of teriparatide in reducing the risk of non-vertebral fragility fractures after a median exposure of 19 months, and showed reversibility of the beneficial effects of teriparatide on femoral neck and hip BMD following discontinuation. Such declines in BMD were prevented by administration of a bisphosphonate and raloxifene after teriparatide treatment, and this response has been observed in both men and women with osteoporosis [57–60].

**Fig. 3:**
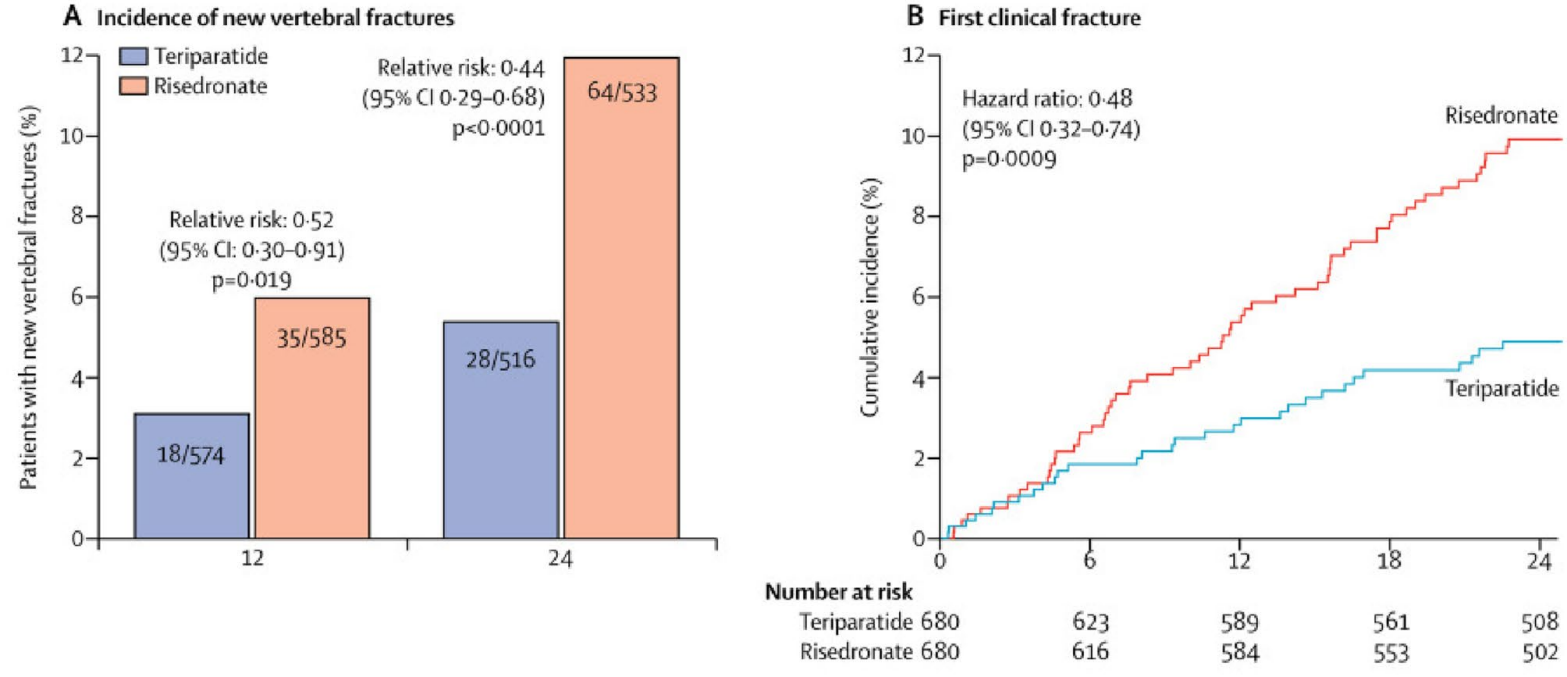
The VERO study of teriparatide vs risedronate; fracture incidence was measured over 24 months. **A** Incidence of new vertebral fractures after 24 months (primary endpoint) and 12 months. At 24 months, new vertebral fractures occurred in 28 (5.4%) of 680 patients in the teriparatide group and 64 (12.0%) of 680 patients in the risedronate group (**B**) Kaplan–Meier estimates of the cumulative incidence of the first clinical fracture, a composite of non-vertebral and symptomatic vertebral fracture. Reproduced with permission from [13]

The VERO study of teriparatide vs risedronate in osteoporosis demonstrated the superiority of the anabolic over the antiresorptive agent in preventing fragility fractures. This was a double-blind, double-dummy trial of postmenopausal women with at least two moderate or one severe vertebral fracture and a BMD T-score ≤ − 1.5. Participants were randomised to 20 µg teriparatide once daily SC plus oral weekly placebo or 35 mg oral risedronate once weekly plus daily injections of placebo for 24 months, with 680 patients in each group. The primary outcome was new radiographic vertebral fractures: at 24 months, new vertebral fractures occurred in 28 (5.4%) of 680 patients in the teriparatide group and 64 (12.0%) of 680 patients in the risedronate group (risk ratio 0.44, 95% CI 0.29–0.68; *p* < 0·0001). Clinical fractures (a composite of non-vertebral and symptomatic vertebral fractures) were also reduced (hazard ratio 0.48, 95% CI 0.32–0.74; *p* = 0.0009), no significant difference in non-vertebral fragility fractures was observed (Fig. 3). The authors concluded that clinicians should consider teriparatide for optimal management of patients with prevalent vertebral fractures—a well-defined high fracture risk group [13].

### Teriparatide plus denosumab, and sequence of agents

Whilst studies combining teriparatide and bisphosphonates have reported no benefit compared to the anabolic agent alone [62, 63], the DATA study combined teriparatide and denosumab for a 24-month period and observed increases in BMD greater than either agent alone [64]. The DATA-Switch extension, in which postmenopausal osteoporotic women were assigned to 24 months of denosumab at the end of the study if they had received teriparatide (so creating a teriparatide to denosumab group), those assigned to denosumab received teriparatide (denosumab to teriparatide group) and those originally assigned to denosumab and teriparatide in combination received an additional 24 months of denosumab alone (combination to denosumab group). In this trial, switching from teriparatide to denosumab, bone mineral density continued to increase, whereas switching from denosumab to teriparatide resulted in progressive or transient bone loss (no fracture data are available) [65].

### Abaloparatide

Abaloparatide is another osteoanabolic drug, also given by subcutaneous injection, designed to have a more rapid onset of action than teriparatide by the strategic insertion of residues into the PTH-related peptide amino-terminal fragment. The resulting peptide is a selective activator of the PTH type 1 receptor signalling pathway and has the ability to produce anabolic effects with modest stimulation of bone resorption in comparison with teriparatide [66]. The ACTIVE trial (Abaloparatide Comparator Trial In Vertebral Endpoints) indicated that abaloparatide treatment for 18 months reduced new morphometric vertebral fractures by 86% and non-vertebral fractures by 43% in comparison with placebo, with rapid separation in non-vertebral fracture risk between the abaloparatide and placebo groups [67].

In the ACTIVExtend trial (determining the efficacy and safety of 18 months of daily subcutaneous abaloparatide compared with placebo (the ACTIVE trial), followed by oral, open-label alendronate for an additional 24 months in women with postmenopausal osteoporosis), abaloparatide followed by alendronate improved bone mineral density and reduced fracture risk, compared with placebo followed by alendronate, with greater benefits the longer alendronate was given. For example, for non-vertebral fractures, the risk reduction was 52% (hazard ratio [HR], 0.48; 95% CI 0.26–0.89). There was also a 58% risk reduction of major osteoporotic fractures (HR 0.42; 95% CI 0.21–0.85) and a 45% risk reduction of clinical fractures (HR 0.55; 95% CI 0.33–0.92) in the abaloparatide followed by alendronate group vs the placebo followed by alendronate group [68], again indicating superiority of using anabolic agents first in sequence in comparison with antiresorptive drugs then anabolic salvage in fracture prevention.

Head to head studies of different PTH analogues are limited [67], however numbers needed to treat were calculated from the pivotal trial for both abaloparatide and teriparatide (as the reciprocal of the absolute risk reductions seen with each agent versus placebo, and clearly with the caveats of separate trial populations) [69]. In order to prevent one new vertebral fracture, 28 women would need to be treated with abaloparatide and 30 treated with teriparatide. To prevent one new non-vertebral fracture, 55 women would need to be treated with abaloparatide and 92 treated with teriparatide. Examining differences in the mechanisms of action and their comparative effectiveness in basic and clinical studies may further guide their clinical application in the future [70].

### Romosozumab

With a completely different mechanism of action, romosozumab is a humanised monoclonal antibody that binds and inhibits sclerostin, and has the dual effect of increasing bone formation and decreasing bone resorption [71, 72]. The FRAME trial enrolled 7180 postmenopausal women between the ages of 55 and 90 years with osteoporosis defined by T-score ≤  − 2.5 at the spine, hip, or femoral neck [73]. Patients were randomised to subcutaneous injections of romosozumab (at a dose of 210 mg in 2 injections) or placebo monthly for 12 months; thereafter, patients in each group received denosumab for 12 months, at a dose of 60 mg, administered subcutaneously every 6 months. After 12 months, a 73% lower risk of vertebral fracture was observed with romosozumab; *p* < 0.001, and a 36% lower clinical fracture risk was seen (*p* = 0.008), but there was no significant difference in the non-vertebral fracture rate. At 24 months, following the transition to denosumab in both groups, the rate of vertebral fractures was 75% lower in the romosozumab group than in the placebo group (*p* < 0.001). In a post hoc analysis of the role of regional background fracture risk on non-vertebral fractures, risk reductions were not observed in Latin America, but romosozumab significantly reduced non-vertebral fracture risk in the rest of the world (42% reduction, *p* = 0.012)[74]. This is probably related to the low risk in these countries given the higher the basal fracture risk, the higher the efficacy of romosozumab [75].

The FRAME extension study demonstrated that one year of romosozumab followed by 2 years of denosumab (compared with 1 year of placebo followed by 2 years of denosumab) led to consistent reductions in fracture risk (new vertebral fracture (relative risk reduction [RRR], 66%; incidence, 1.0% versus 2.8%; *p* < 0.001), clinical fracture (RRR, 27%; incidence, 4.0% versus 5.5%; *p* = 0.004), and non-vertebral fracture (RRR, 21%; incidence, 3.9% versus 4.9%; *p* = 0.039) and ongoing BMD gains. At 36 months, in the group that received romosozumab followed by 2 years of denosumab, mean T-scores improved from − 2.7 to − 1.5 in the spine and from − 2.5 to − 2.0 in the total hip. The proportion of participants who had a T-score in the osteoporosis range decreased from 63% at baseline to 20% at 3 years in the spine and from 53 to 14% in the total hip [76].

In an analysis of treatment response, BMD gains of ≥ 6% in the spine were achieved by 68% of participants who received romosozumab versus 6% in those on placebo at the 1-year timepoint, and at the total hip, BMD gains of ≥ 6% were seen in 47% of romosozumab-treated versus 3% of placebo-treated patients; such BMD gains were reflected in lower fracture rates. The magnitude of the BMD gains was large in the romosozumab/denosumab treatment sequence; at 2 years such gains were similar to the BMD gains observed with 7 years of denosumab alone in the Freedom/Freedom Extension study [77, 78].

In the ARCH study, romosozumab was compared directly to the antiresorptive bisphosphonate, alendronate. 4093 women, age range 55–90 years, with prevalent osteoporotic fracture were enrolled and randomised to receive romosozumab 210 mg subcutaneously once monthly or alendronate 70 mg once weekly orally for 1 year in a double-blind fashion. All participants subsequently received open-label alendronate for the remainder of the study with a time-to-event design, which had a median treatment period of 2.7 years (33 months). Romosozumab followed by alendronate was more effective than alendronate in preventing vertebral fractures at 24 months, (risk ratio 0.52; incidence 6.2% versus 11.9%; *p* < 0.001), and clinical fractures at the time of primary analysis, co-primary endpoints (risk ratio 0.73; incidence 9.7% versus 13.0%; *p* < 0.001). In addition, the risk of non-vertebral fractures was lower by 19% in the romosozumab-to-alendronate group than in the alendronate-to-alendronate group (incidence 8.7% vs 10.6%; *p* = 0.04), and the risk of hip fracture was lower by 38% (incidence 2.0% vs. 3.2%; *p* = 0.02). Gains in BMD were substantially higher in patients who received romosozumab compared with alendronate in year 1. In the romosozumab group the BMD gains were very similar to those seen in FRAME. In the ARCH trial, BMD increased further after the transition to alendronate; however, the BMD gains at 36 months were not as impressive as those seen in FRAME when women transitioned from romosozumab to denosumab [14].

Reversal of the beneficial effects of romosozumab on cessation of therapy (2 years denosumab and switched to placebo), and prevention of BMD loss at the lumbar spine and hip by administration of denosumab was demonstrated by McClung et al. Women receiving romosozumab who transitioned to denosumab continued to accrue BMD, whereas BMD returned toward pretreatment levels with placebo [79]. McClung et al. also demonstrated that after a second course of romosozumab, administration of an antiresorptive (intravenous zoledronate) also maintained the robust BMD gains observed with romosozumab treatment, though without antiresorptive administration, such beneficial effects were lost [80].

It is of interest that the effects of romosozumab are greater the higher the fracture probability at baseline [75], a phenomenon which is not seen with teriparatide [81, 82]. This makes romosozumab of particular relevance in patients at very high fracture risk.

An important and as yet not completely resolved consideration with the use of romosozumab is the apparent increased risk of cardiovascular adverse outcomes in the treatment group in the ARCH trial. In post hoc analyses of the composite outcome of non-fatal MI, non-fatal stroke and cardiovascular death plus heart failure and non-coronary heart disease, the incidence was 2.0% in the romosozumab group and 1.1% in the alendronate group (HR 1.7, 95% CI 1.1–2.6). A similar signal was not seen in the FRAME trial and is currently subject to ongoing post marketing surveillance studies [83].

## Choice of anabolic agent

The ESCEO working group agreed that it is difficult to differentiate between the available anabolic agents as head-to-head comparisons are limited to two studies [67, 84], hence such recommendations are outside the scope of this document. Therefore, recommendations apply to anabolics as a class rather than as individual agents.

An important consideration for the implementation of the recommendations made in this position paper, of “anabolic first” in patients at very high risk of fracture, is that the availability of any particular medication will likely be different by country and also potentially by healthcare providers within countries. For example, at the time of writing, teriparatide is widely available globally, whereas abaloparatide is not licensed in Europe. In the UK, whilst a medication may be licensed by the regulatory authorities, its use in the National Health Service may be further adjudicated by the National Institute for Health and Care Excellence (NICE). Historically, as with many healthcare systems, the available anabolic, teriparatide, was specifically positioned for those patients with severe osteoporosis who have failed other therapies or with multiple subsequent fractures; thus a paradigm change to initial treatment with anabolics will in many cases require alterations to national guidance. Reassuringly, continuing the example of the UK, the earlier shift from BMD to absolute fracture risk based on FRAX has demonstrated that clinician led guidance, through the UK National Osteoporosis Guideline Group (produced as a response to the impractical NICE approach) has subsequently become NICE accredited and incorporated into NICE quality standards [85, 86]. The move to remission and maintenance has already been achieved in inflammatory disease such as rheumatoid arthritis, and the ability of the field to change the paradigm, followed by corresponding alteration to national guidance and policy, should challenge all of us to move our clinical approach forward in a way that best serves the needs of our most vulnerable patients.

## Duration of anabolic agent

### Teriparatide

The duration of treatment with teriparatide was initially limited by safety concerns; preclinical evidence suggested a potential association between teriparatide and osteosarcoma. Specifically, in rat studies, a dose-dependent increase in the risk of osteosarcoma incidence was observed after teriparatide administration, given since weaning [87]. Consequently, prescribing information for teriparatide includes a warning about a potential risk of osteosarcoma and precautions against use of the product for patients with risk factors for osteosarcoma (e.g. Paget's disease of the bone, unexplained increase in alkaline phosphatase, open epiphyses, prior radiation therapy). The current prescribing information recommends that lifetime treatment with teriparatide should be limited to a maximum duration of 2 years. Reassuringly, a recently published post marketing surveillance study of US cancer registries showed that the incidence of osteosarcoma associated with teriparatide use during the 15-year surveillance period was no different than would be expected, based on the background incidence rate of osteosarcoma [88].

A 2-year treatment course of teriparatide was shown to be appropriate in the EUROFORS study. In this prospective, randomised, controlled, 2-year study, BMD effects and clinical safety of three follow-up treatments (anabolic with teriparatide, antiresorptive with raloxifene, or no active treatment) after 1 year of teriparatide were compared. BMD was shown to increase progressively over the 2-year teriparatide therapy (increasing spine BMD by 10.7%), whereas patients receiving raloxifene in year 2 had maintenance, but no further increase in spine BMD from year 1 (change from baseline, 7.9%), and patients receiving no active treatment had a BMD decrease of 2.5% in year 2 (change from baseline, + 3.8%) [59]. Histomorphometric data support this 2-year treatment approach, demonstrating an increase in the mineralising surface available in the endocortical, periosteal and intracortical compartments [89], in addition to the trabecular network [90].

Such changes in BMD and histomorphometry are reflected by decreases in fracture risk over time: Lindsay et al., using post hoc analysis of data from the Fracture Prevention Trial, demonstrated that compared with placebo, the relative hazard for non-vertebral fragility fractures decreased by around 7% for each additional month of teriparatide treatment [91]. Similarly, in the VERO study of teriparatide versus risedronate, a continuous decrease in the rate of clinical fractures over a 2-year time period was observed in the teriparatide group [13].

To summarise, the evidence suggests that 24 months is a reasonable time frame over which to recommend teriparatide use. Few studies exist, however, testing a longer treatment duration. In one study of teriparatide versus alendronate in glucocorticoid induced osteoporosis, an increase in lumbar spine and hip BMD was observed over a 36-month period, with fewer new vertebral fractures in the teriparatide group than in patients treated with alendronate [92]. Bone turnover markers (BTMs) in the same study provide an insight into the optimal duration of teriparatide use. An increase in procollagen type I N propeptide (PINP) and osteocalcin (bone formation markers) was observed in the first six months of treatment, then there was a slight decrease over time. C-terminal cross-linking telopeptide of type I collagen (CTX-I), indicating bone remodelling, also increased over six months and then decreased over time almost back to baseline by 18 months [92]. This pattern of waning of the effect of teriparatide on markers of bone formation and remodelling has been observed in various different trials, and in one study inhibitors of the WNT pathway (Dickkopf-1 (DKK1) and sclerostin) were also measured over and 18-month period of teriparatide use, with an increase and then levelling off in DKK-1 over time. Therefore, changes in the regulation in bone physiology and the Wnt pathway could be part of the explanation of waning effects of teriparatide beyond 18 months–2 years [93].

### Abaloparatide

The ACTIVE trial compared abaloparatide with teriparatide. As similar concerns regarding osteosarcoma risk were applied to abaloparatide (given its similar mechanism of action); the trial thus focused on the short-term use of this drug [67]. The BMD changes demonstrated a slightly greater effect of abaloparatide than teriparatide at the total hip, femoral neck and lumbar spine. When observing BTMs (CTX-I and PINP) the profiles of changes were quite different between the two drugs, a steep increase in the first three months followed by a slow decline to baseline was observed with abaloparatide, compared to a slower rise reaching a peak at 6–12 months with teriparatide. Comparing the uncoupling index (the balance between bone formation and resorption markers) between abaloparatide and teriparatide, there was only a slightly better uncoupling index at 1 month in abaloparatide but the profile was quite similar over time (18-month duration of the study). A strong association with the ratio of PINP at 3 months versus baseline with lumbar spine BMD was observed in both drugs (slightly stronger in abaloparatide) indicating that the rapid stimulation of bone formation with a high uncoupling index in the first few months of treatment is particularly important, supporting short-term use of this bone-forming agent [94].

### Romosozumab

Studies evaluating BTMs in Phase 2 studies of romosozumab also show a rapid increase in markers of bone formation early in treatment, but with an uncoupling effect between formation and resorption. A rise in PINP (even steeper than observed with abaloparatide) followed by a fall back to baseline within the first 6 months of treatment is observed, alongside a sharp drop in bone resorption (CTX-I) on starting the agent, returning to baseline at 3–6 months, with both markers remaining below baseline at month 12. This suggests that after 1 year, romosozumab is a moderate bone remodelling inhibitor, rather than a potent bone-forming agent. Following transition to denosumab at 24 months, in this study, there was a further rapid decrease in bone remodelling [79]. Regarding romosozumab BMD data, this is slightly different, showing more sustained increases; there was an increase in BMD at the lumbar spine and total hip up to 24 months (with no effect at the radius). On switching to denosumab there was a further steady increase in BMD at both the lumbar spine and total hip. The majority of gains in BMD are observed in the first year, however, supporting this duration of use.

## Bone turnover markers in treatment stratification

When a choice of sequential treatments is available to a clinician, it may be possible to use BTMs to aid medication decisions. Studies have demonstrated that rapid bone loss is associated with increased levels of BTMs [95]. It is also well established that elevated BTMs are associated with increased fracture risk, as demonstrated by a meta-analysis performed by the IFCC-IOF committee on BTMs, such that for each SD increase in PINP there was around a 23% increased risk of fracture; this was also true for CTX-I (18% increased risk per SD) [96]. However, one problem in the interpretation of BTMs in patients with osteoporosis is that patients who have recently fractured have high levels of such markers and these effects can persist for at least 4–6 months after the fracture, in many cases longer [97, 98]. Nonetheless, as previously discussed, there is substantial evidence that patients who have recently fractured are at high imminent risk of further fracture.

There is some evidence for a BMD independent predictive value of high BTMs as shown by the EPIDOS prospective study; therefore, patients with both low BMD and high BTMs are likely to be at very high risk of incident fracture [99]. However predictive capacity within a population has limitations when applied to an individual because of the substantial inter- and intra-individual variation in BTM. As discussed below, recent approaches have applied a “least significant change” approach to the use of BTM in monitoring treatment but the incorporation of BTM into prediction of fracture risk at the individual level remains a challenge.

In the future, novel biochemical markers such as high sensitivity CRP (hs-CRP) may be useful: in a recent meta-analysis of 10 studies, the RR for fracture in a comparison of the top third group to the bottom third group of hs-CRP was 1.54 (1.18–2.01), though these findings cannot be applied to an individual patient [100]. Similarly, uric acid has been shown to be predictive of fracture, with an increased serum uric acid level associated with a lower risk of fracture [101]. Higher cystatin C has been associated with higher risk of hip fractures in older women, independent of renal function [102]. Other markers may be of interest for future prospective studies including periostin, cathepsin K, osteoprotegerin (OPG), RANKL, DKK-1, sclerostin, FGF-23, Klotho, and of course miRNAs (but here we need to define the most interesting miRNAs) [103], with another novel marker, high sphingosine-1-phosphate, having been shown to be a risk factor for osteoporotic fracture independent of FRAX probability, in an Asian population [104].

Finally, reductions in BTMs in patients without a recent fragility fracture using antiresorptive therapy is known to reflect good compliance and treatment response [105]. As outlined by the ESCEO and IOF algorithm, BTMs are recommended to be checked at baseline and 3 months after starting therapy, with responders to antiresorptive considered to be those who show changes in BTMs that exceed the least significant change (56% decrease for CTX-I and 38% decrease for PINP) [43]. If at three months a decrease is not seen, it is recommended that adherence is discussed with the patient, and if they are adhering well, a treatment change may be considered, though the magnitude of BTM response may differ between therapies. In implementing such findings, further work remains to optimise the utility of BTMs in choosing between sequential therapies, though the knowledge that high levels of BTM in concert with low BMD constitutes high fracture risk is important.

## Long-term treatment: cycling of anabolic/antiresorptive therapies?

In patients at high fracture risk, particularly younger patients, it is possible that the sequence of bone-forming agent to antiresorptive therapy might be repeated, or that shorter sequences of anabolic followed by antiresorptive therapies may be more efficacious. Cyclic administration of teriparatide alone has not been shown to increase BMD more than standard daily therapy. In a study by Cosman et al., teriparatide was given for four 3-month cycles, each followed by 3 months off, and compared to daily teriparatide for 24 months, in both alendronate naïve and women on alendronate. In the women on alendronate, cyclic teriparatide over 2 years improved BMD similarly to daily treatment in women who remained on alendronate (despite only 50% of the teriparatide dose), but in treatment naïve women there did not appear to be a BMD advantage to cyclic administration [106]. Cosman et al. further hypothesised that, because denosumab is a potent antiresorptive agent with a rapid off-effect, it might be the optimal agent to help maximise bone gains with cyclic teriparatide. In a 3-year study, 70 postmenopausal women with osteoporosis were randomised to 18 months of teriparatide followed by 18 months of denosumab (standard) or three separate 12-month cycles of 6 months of teriparatide followed by 6 months of denosumab (cyclic) [107]. The findings suggested a slight benefit of the cyclical approach at 18 months, particularly in the cortical skeletal sites (particularly the 1/3 radius), but the cyclic regimen did not demonstrate a benefit at 36 months [107]. The authors concluded that this cyclic approach could be useful in patients at the highest risk of imminent fracture, but further studies of short sequences of cyclic therapy are required, covering the different anabolic agents, with assessment of BTMs, bone structural and densitometric evaluation and assessment of fracture prevention.

The decision-making process on when (and if ever) to stop antiresorptive therapy in a patient who has received a prior anabolic agent, is often complex, and of course only minimally informed by the very limited evidence base. It is not known whether discontinuation of the second, antiresorptive agent (usually a bisphosphonate, SERM or denosumab) induces the same effect as for the second drug used alone. In a recent narrative review of available clinical trials in which therapies were discontinued and followed up for a year or more, Elbers et al. demonstrated 0.4% or lower decrease in femoral neck BMD after 1 year of discontinuation of therapy in both previously alendronate- and zoledronate-treated patients; hence in these patients a discontinuation of up to a year may be acceptable [108]. In the other reported agents (risedronate, ibandronate, raloxifene, teriparatide, denosumab and romosozumab) this percentage of bone loss at the femoral neck and total hip was at least 1%, with the largest decrease in BMD after discontinuation of denosumab and romosozumab, providing further support for the importance of continuation of antiresorptive agents after both agents [108].

The consensus of the group was that, if the patient remains at high risk or very high risk of fracture, it is likely that a patient will need prolonged antiresorptive therapy after anabolic treatment. However, if a patient reaches a BMD such that they are no longer at high or very high risk it may be possible to stop treatment for a short window of a maximum of a couple of years, though not if denosumab is the antiresorptive agent in use due to the risk of rebound vertebral fractures. In the case of denosumab, there is some evidence that an infusion of 5 mg zoledronate helps to reduce the rebound loss in BMD on stopping denosumab, and helps prevent vertebral fractures [109, 110]. Studies suggest that a longer duration of denosumab treatment, of more than 6 years, puts an individual at greater risk of fracture on stopping [111, 112], as does the presence of prevalent fractures [113], so supporting the need for a careful clinical approach in denosumab cessation [113, 114].

## Medication adherence

It is widely recognised that patient adherence to oral bisphosphonates for osteoporosis, and the strict conditions for taking them, is poor. In a systematic review of 89 observational studies, the mean persistence of oral bisphosphonates for 6 months, 1 year and 2 years ranged from 34.8% to 71.3%, 17.7% to 74.8% and 12.9% to 72.0%, respectively, indicating that persistence and adherence reduced notably over time [115]. Across 27 different retrospective studies, non-adherence is associated with a 29% increased risk of osteoporotic fractures, and non-persistence with a 40% increased risk of osteoporotic fractures (over a 104–159-week period) [116]. Not only does poor adherence have a huge clinical burden, it also has an economic impact: a study based on Irish data demonstrated around a 50% reduction in the potential benefits (quality-adjusted life years (QALY)) gained with real-world adherence compared with patients with good adherence observed in clinical trials, and a doubling of the cost per QALY gained from these medications [117].

Poor adherence to antiosteoporosis therapy has been attributed to a variety of factors, particularly when oral bisphosphonates are considered, but also in the case of parenteral therapies including anabolic agents [118]. These include condition-related factors, such as polypharmacy, older age of patients and lack of disease awareness, medication-related factors such as the frequency of dosing and side effects, health system-related factors such as care across different specialties and poor patient education, and economic factors such as poor insurance coverage [119]. A variety of studies and experts (including an IOF-ESCEO working group) have evaluated patient preferences for osteoporosis drug treatment, in order to design antiosteoporosis treatments which may counter this adherence and persistence problem [120]. Whilst findings across studies are heterogenous, and differ between countries, in general the important attributes of a treatment to encourage adherence and persistence are its effectiveness, a good side-effect profile, ease of administration, lower costs, lower treatment duration and the availability of patient-support programmes [121–126]. Wider use of treatments with preferable characteristics (which may be true of sequential treatments over antiresorptives alone) might lead to greater antiosteoporosis medication adherence, though there are not yet any clinical trials to support this notion.

## Cost-effectiveness of sequential treatments

In the context of spiralling fracture-related costs worldwide, health economic evaluations of treatments have become increasingly important. Such evaluations help to support priority setting in healthcare, aiding decision makers in the efficient allocation of limited healthcare resources in the ageing populations seen throughout the world. In the field of osteoporosis, the availability of newer, more expensive antiosteoporosis therapies, including abaloparatide and romosozumab, and interest in the use of sequential therapies, has brought the importance of cost-effectiveness analysis into sharper focus.

In 2021, Li et al. published an updated systematic review of the cost-effectiveness analyses of drugs for osteoporosis, using the guidelines set out by ESCEO-IOF for the conduct and reporting of economic evaluations for osteoporosis [127, 128]. This review covered studies published between 1 July 2013 and 31 December 2019, and covered the cost-effectiveness of sequential therapy in the form of a bone-forming (anabolic) agent (i.e. abaloparatide and original/branded teriparatide), followed by an antiresorptive. Three studies were identified (all in postmenopausal women in the US at increased risk of fracture), comparing abaloparatide followed by alendronate versus teriparatide followed by alendronate (Le et al., 2019) [129], sequential teriparatide/alendronate versus alendronate alone (Mori et al., 2019) [130] and sequential abaloparatide/alendronate versus teriparatide/alendronate (Hiligsmann et al., 2019) [131]. These studies used quality-adjusted life years (QALY) as their outcome measures as recommended, in addition to either lifetime (Mori and Hiligsmann) or 10-year time horizons (Le). These studies had mixed results (partly due to different drugs, in different age groups, with different BMD T-scores at baseline); Hiligsmann and Mori both showed that abaloparatide followed by alendronate was dominant (better outcomes, lower costs) when compared with teriparatide followed by alendronate [130, 131]. Hiligsmann also showed that abaloparatide followed by alendronate, when compared with no treatment, was cost saving or cost effective in different populations [131]. Conversely, Le et al. found that abaloparatide or teriparatide followed by alendronate was not cost effective when compared with a placebo followed by alendronate [129]; when Mori compared sequential therapy (teriparatide followed by alendronate) with alendronate alone at different ages and economic perspectives, sequential therapy was not shown to be cost effective [130]. These studies found that the high costs of abaloparatide and teriparatide largely affected incremental cost-effectiveness ratios (ICERs) when these treatments were compared with no treatment, placebo or alendronate alone. Generic/biosimilar teriparatide (if at 65–85% of the brand cost) can finely alter the balance, bringing the ICERS of sequential teriparatide/alendronate to below the willingness-to-pay (WTP) threshold of $150,000 US/QALY [130]. The systematic review commented on the limited number of studies of cost-effectiveness of sequential therapy conducted to date, particularly in very high fracture risk patients, and the need for further research in this area.

Since this review including publications up to December 31st 2019, other studies have suggested that sequential treatment may be cost effective [133–136]. Hiligsmann et al. limited their population to women with severe osteoporosis in the US (50–80 years with BMD T-score ≤ -3.5 and without a history of prior fracture, or with a T-score of − 2.5 and − 3.5 with a history of ≥ 1 osteoporotic fracture); sequential abaloparatide (18 months) followed by alendronate (5yrs) was compared to generic alendronate monotherapy. Sequential abaloparatide/alendronate (threshold of $150,000 US/QALY) was found to be cost effective in women ≥ 60 years. Soreskog et al., in a study in the UK considering a 1-year bone-forming agent, followed by antiresorptive for 4 years (versus antiresorptive monotherapy) in 70 year old women with a T-score of − 2.5 and recent major osteoporotic fracture, found that the cost per QALY gained in the base-case setting was estimated at £34,584 [133]. Soreskog also modelled a Swedish population in which patients aged 74 years with a recent major osteoporotic fracture were treated with romosozumab for 12 months followed by alendronate for up to 48 months or alendronate alone with a maximum duration of 60 months. Sequential romosozumab-to-alendronate treatment was associated with 0.089 additional QALYs at an additional cost of €3002 compared to alendronate alone, resulting in an ICER of €33,732. At a Swedish reference willingness-to-pay per QALY of €60,000, the authors concluded that romosozumab-to-alendronate had a 97.9% probability of being cost effective against alendronate alone. Mori et al., studying a Japanese population of older osteoporotic women with prior vertebral fractures compared sequential teriparatide (2 years) followed by alendronate for 8 years, versus alendronate monotherapy. They found, again, that cost-effectiveness was very sensitive to treatment acquisition costs, only becoming cost effective with price discounts to teriparatide of 85%, 50%, and 15% at ages 70, 75, and 80 years, respectively, compared to the current biosimilar cost [135].

To conclude, there is emerging evidence for the cost-effectiveness of sequential treatment in osteoporosis, with bone-forming agents followed by antiresorptive agents being cost effective compared to antiresorptive alone. However, this depends upon treatment acquisition costs, and is limited to patients at very high risk of fractures; the exact fracture probability at which sequential treatment regimen is cost effective needs to be ascertained, and of course is likely to vary between healthcare systems and countries. Another recommendation for future research is to determine the maximum cost that a sequential treatment combination can hold to be cost effective within a population at a defined risk of fracture. Thus, as a result of differences in epidemiology, costs and drug prices between countries, local economic evaluation would be needed.

## Clinical approach

Our ability to target patients with osteoporosis at the highest risk of fracture for the most effective antiosteoporosis treatments has been identified as a clinical priority for many years and has been cited as one important strategy towards closing the osteoporosis treatment gap. We can conclude from the evidence presented above that, in patients at very high risk of fracture, sequential therapy (commencement of treatment with a bone-forming agent, followed by an antiresorptive to maintain the gains in bone mineral density), appears to be an appropriate strategy to achieve a rapid and sustained reduction in fracture risk.

**Fig. 4:**
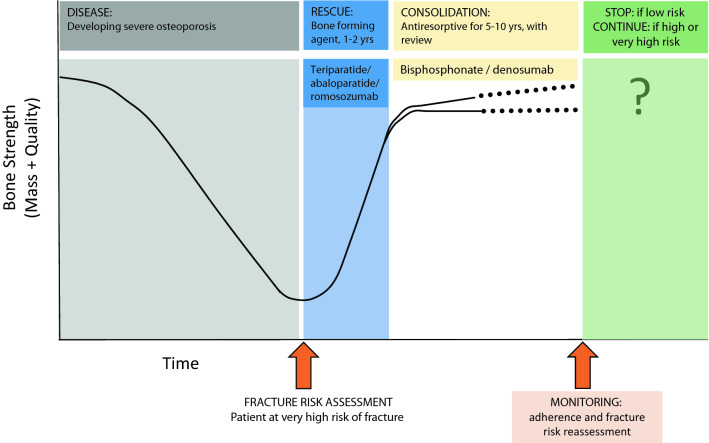
Outline of a recommended approach to sequential therapy: in a patient with severe osteoporosis at high imminent risk of fracture following fracture risk assessment, a bone-forming agent for 1–2 years is recommended (duration according to prescribing guidelines). Following this, bone-forming therapy, a consolidation period of antiresorptive therapy (such as a bisphosphonate or denosumab) is recommended. Monitoring, including assessment of treatment adherence and reassessment of fracture risk, is required

We, therefore, propose a pragmatic approach to patients at very high fracture risk as outlined in Fig. 4. If an individual is found to be at very high fracture risk (which may be assessed through a variety of risk assessment modalities) they will be recommended for initial therapy with a bone-forming agent. Currently available such agents include teriparatide, abaloparatide and romosozumab (dependent on country). These should be used for the duration recommended by prescribing guidelines. It is expected that an individual’s BMD improves rapidly over this period, with a consequent reduction in fracture risk. Following this bone-forming therapy, a consolidation period of antiresorptive therapy (such as a bisphosphonate or denosumab) is recommended, leading to either stabilisation, or gradual improvements in bone quality. Monitoring, including assessment of treatment adherence and reassessment of fracture risk, is required, to ensure that the optimal antiresorptive therapy has been chosen for the patient with minimal adverse effects, and to provide support for the achievement of maximal adherence. Longer term, given the very high fracture risk starting point for this patient population, in the vast majority of cases, consideration of the need for antiosteoporosis medication will continue to be relevant indefinitely.

## Conclusion

We have already witnessed a paradigm shift towards early optimised treatment to achieve remission and maintenance in other areas of musculoskeletal medicine, such as in the management of inflammatory arthritis. There is now an urgent call to the osteoporosis field to alter our treatment paradigm towards anabolic therapies first in our highest fracture risk patients. Whilst there may be barriers in terms of existing national policy and availability of medications, the consensus view from this ESCEO working group strongly supported this direction as the most appropriate way in which we can make the greatest difference to the care of our most vulnerable patients.
